# Generalizability of heat-related health risk associations observed in a large healthcare claims database of patients with commercial health insurance

**DOI:** 10.1097/EDE.0000000000001781

**Published:** 2024-08-09

**Authors:** Chad W. Milando, Yuantong Sun, Yasmin Romitti, Amruta Nori-Sarma, Emma L. Gause, Keith R. Spangler, Ian Sue Wing, Gregory A. Wellenius

**Affiliations:** 1 Department of Environmental Health, Boston University; 2 Department of Earth and Environment, Boston University; 3 Center for Climate and Health, Boston University School of Public Health

**Keywords:** Heat-related health impacts, time-stratified case–crossover, Optum Labs Data Warehouse, generalizability

## Abstract

**Background:**

Extreme ambient heat is unambiguously associated with higher risk of illness and death. The Optum Labs Data Warehouse (OLDW), a database of medical claims from US-based patients with commercial or Medicare Advantage health insurance, has been used to quantify heat-related health impacts. Whether results for the insured sub-population are generalizable to the broader population has to our knowledge not been documented. We sought to address this question, for the US population in California from 2012 to 2019.

**Methods:**

We examined changes in daily rates of emergency department (ED) encounters and in-patient hospitalization encounters for all-causes, heat-related outcomes, renal disease, mental/behavioral disorders, cardiovascular disease, and respiratory disease. OLDW was the source for health data for insured individuals in California, and health data for the broader population were gathered from the California Department of Health Care Access and Information (HCAI). We defined extreme heat exposure as any day in a group of 2 or more days with maximum temperatures exceeding the county-specific 97.5^th^ percentile and used a space-time-stratified case–crossover design to assess and compare the impacts of heat on health.

**Results:**

Average incidence rates of medical encounters differed by dataset. However, rate ratios for ED encounters were similar across datasets for all causes (ratio of incidence rate ratios (rIRR) = 0.989; 95% confidence interval (CI) = 0.969, 1.009), heat-related causes (rIRR = 1.080; 95% CI = 0.999, 1.168), renal disease (rIRR = 0.963; 95% CI = 0.718, 1.292), and mental health disorders (rIRR = 1.098; 95% CI = 1.004, 1.201). Rate ratios for inpatient encounters were also similar.

**Conclusions:**

This work presents evidence that OLDW can continue to be a resource for estimating the health impacts of extreme heat.

## Introduction

Extremely hot days are unambiguously associated with higher risk of illness and death.^1,2^ In the US, this association has been documented using various large healthcare claims datasets. Among the US Medicare population (adults 65 and older), extreme heat has been associated with increased hospital admissions for a range of diagnoses including heat stroke, fluid and electrolyte disorders, and renal failure.^3–5^ Claims datasets for specific US states have been used to associate extreme heat with emergency room visits for all-cause and mental-health related outcomes.^6–8^ Hess et. al. (2014) used a nationally representative dataset of annualized summertime emergency department visits to show the adverse impact of heat on health across the US.^9^ Bernstein et. al. (2022) used daily counts of individuals admitted to children’s hospitals in the US to show the impact of heat on adolescent health.^10^ Commercial insurance claims datasets, like those in the Truven Health MarketScan® Research dataset or Optum Labs Data Warehouse (OLDW), have also been used to document the association between high warm-season ambient temperatures (i.e., “heat”) and risk of emergency department visits for a range of physical and mental health conditions.^11–15^

Although the conclusions of these heat and insurance claims studies are similar, studies vary in terms of exposure metrics, temporality, and the strengths and potential limitations of their health outcome data. This leads to heterogeneity in the interpretation and magnitude of effect estimates and raises several overarching questions. The common underlying population-level association between days of extreme heat and changes in human health remains unknown. This association may be difficult to discern in the absence of a nationally representative dataset. Large insurance claims datasets can stand in as proxies for a national dataset but may over- or under-represent different segments of the population. It is therefore essential to assess whether heat–health associations generated using insurance claims datasets are generalizable to the general population.

In this study we address the question of generalizability of heat and health associations created using commercial claims data by leveraging the overlap in space and time across two datasets of hospital-patient encounters. The first dataset – the OLDW – contains healthcare claims data for millions of individuals of all ages with commercial or Medicare advantage health insurance, representing an estimated 6.4% of the US population as of July 2015.^15^ Notable strengths of the OLDW include its very large size, inclusion of patients of all ages living almost anywhere in the contiguous US, geographic resolution down to the zip code level, ability to follow individuals over time, detailed information on all medical encounters, and a high refresh rate (data are considered complete with a 6-month lag) that facilitates timely event study health impact assessments.^13^ The principal limitation of the OLDW in the context of environmental health research is its restriction to individuals with commercial or Medicare Advantage health insurance. Individuals with health insurance typically have higher rates of healthcare utilization but are also likely healthier than individuals without health insurance^16^ and potentially more protected from extreme weather,^17^ raising the possibility that heat–health estimates derived from the OLDW may underestimate the health impacts of heat in the general population.

The second health outcomes dataset utilized in this study was hospital discharge data for the general population obtained from all licensed hospitals in California reporting data to the state’s Department of Health Care Access and Information (HCAI).^18^ This dataset has the benefit of containing information from both insured and non-insured individuals in the state of California and serves to represent heat–health impacts among the general population.

We used a space-time stratified case–crossover study design to compare heat–health associations estimated from the OLDW dataset residing in the state of California from 2012 to 2019 with associations estimated from HCAI discharge dataset for the same time period. California is an apposite environment to test the generalizability of OLDW-derived heat–health associations because of the within-state heterogeneity in contextual factors, climate zones, sociodemographics, and urban/rural populations. We first compared demographic characteristics of patients and incidence rates of emergency department (ED) visits and inpatient hospitalizations in the two datasets. Next, we standardized daily counts of ED visits and inpatient hospitalizations in the OLDW dataset according to the age, sex, and county distribution of the population of California for the same years.^18^ Finally, we calculated incidence rate ratios (IRR) between markers of extreme heat and healthcare utilization in the OLDW and the HCAI datasets, and compared IRRs via a ratio of incidence rate ratios (rIRR) to assess the generalizability of the former results.

## Methods

### Study populations

For this study we focused on persons aged 18 years or older residing in California during the warm seasons (May 1 through September 30) of 2012 to 2019. Daily person-time at risk for individuals in the OLDW and HCAI datasets was estimated in strata by age group (18-24, 25-34, 35-44, 45-54, 55-64, 65-74, 75+), sex (male or female), and county of residence. For OLDW, we assigned the daily person-time at risk in each stratum as the average number of OLDW enrollees in each stratum during that day’s month. The population reflected in the HCAI hospital record data were inferred based on population count data for the state of California obtained from the Centers for Disease Control and Prevention (CDC).^19^ CDC data were available in 5-year age groups and did not include a split at age 18. Therefore, for the HCAI dataset, counts for persons aged 18 or 19 years were approximated in each county by taking a random sample of 40% of persons with ages between 15 and 19 years (2 of 5 years between 15 and 19) and assigning them to the age group for 18 to 25.

### Outcomes

We analyzed patterns in emergency department (ED) visits and inpatient hospitalizations for all causes, as well as those known to be exacerbated by heat exposure, including renal disease, heat-related illnesses, mental/behavioral disorders, cardiovascular disease, and respiratory disease (eTable 1). Patient records were retrieved from OLDW and a comparison dataset obtained from the HCAI which includes outcomes from all patients who received care at general acute care service centers in California (which includes essentially all hospitals and freestanding ambulatory clinics, and excludes military facilities).^18^ Although both datasets derive from the underlying population of California residents, anonymization and data licensing restrictions precluded their linkage (see explanation in eText 1). In the remainder of this manuscript, we refer to ED visits and inpatient hospitalizations as ED and inpatient “encounters,” and the OLDW and HCAI as “datasets” (rather than as “populations”). Analyses of the OLDW data were approved by the Boston University Medical Campus Institutional Review Board (IRB). Analyses of the HCAI data were approved by the Boston University IRB and the CA Committee for the Protection of Human Subjects.

In the OLDW dataset, claims for ED and inpatient encounters were identified by revenue codes, Current Procedural Terminology (CPT) codes, and place of service code (eFig. 1). We grouped claims into encounters by first claim date: any claims that occurred within 1 day of a previous claim for the same person were treated as the same encounter. We aggregated encounters into groups defined by encounter start date, encounter type (ED or inpatient), age group, sex, and county of patient residence. Information on other individual-level demographic characteristics such as income, race, or ethnicity, was not available. We categorized encounters containing only ED claims as an ED encounter; we categorized encounters including both ED and inpatient claims as inpatient encounters – we chose this categorization to align with how ED and inpatient encounters are recorded in the HCAI dataset.

HCAI data contain records of ED and inpatient encounters at essentially all hospitals in California. Individual-level encounters included encounter start date, county of patient residence, patient age and sex, and primary and secondary diagnosis codes; these were aggregated in a manner similar to that described for OLDW. We did not have access to expected payer records in this dataset, which precluded stratification of the HCAI dataset into individuals with and without health insurance.

### Exposure

We estimated exposure to ambient extreme heat using population-weighted temperature metrics from the Parameter-elevation Regressions on Independent Slopes Model (PRISM),^20^ a spatiotemporal gridded meteorological dataset with approximately 4km horizontal grid spacing.^21^ Drawing on prior research,^22^ we defined a county-specific heatwave as a sequence of 2 or more consecutive days during which maximum temperatures exceeded the year-round 97.5^th^ percentile of county-specific daily maxima over a baseline period of 1999 to 2010 (method further described in eText 2). Within every heatwave spell that met this criteria, all constituent days were assigned as heatwave days and were matched to patient-hospital encounters according to the date of first claim.^22^ The latter constituted our main exposure variable, which accounted for between 4.3 and 10.9% of days during each summer (eTable 2). In sensitivity analyses, we compared these results to models using a simpler exposure metric: a binary indicator of whether daily maximum temperature exceeded the local 97.5^th^ percentile; by comparison 4.6 to 11.1% of days met this criterion.

### Analytical Approach

For both the OLDW and HCAI datasets, we created time-series of daily ED or inpatient encounters within each age-sex-county-year stratum. For the OLDW data, we used CDC data to standardize these daily encounters by re-weighting the counts in each stratum so that the OLDW dataset more closely reflected the population of the state of California in each year (see sample calculation in eText 3), following recent approaches.^23^

We compared incidence rates (IR) expressed as encounters per million person–days. We then applied a space-time-stratified case–crossover design to empirically estimate the association between markers of extreme heat and rates of ED or inpatient encounters expressed as incident rate ratios (IRR).

### Heat–health associations

We used a conditional quasi-Poisson regression,^24,25^ with stratum (*s*) for county, year, month, and day-of-week. Conditional Poisson models provide computational gains over traditional unconditional models in regressions with many strata,^26,27^ and have been used in many case–crossover studies of the health impacts of heat.^26,28–30^ See Armstrong and Gasparrini (2014) for an elaborated discussion of the conditional Poisson modeling setup.^24^ A quasi-Poisson distribution was chosen to account for overdispersion. The outcome was the daily number of ED or inpatient encounters, *N*_*t*,*s*_ for day *t* in strata *s*. Our predictor of interest was a binary heat wave day indicator (*hw*). Additional statistical controls included a binary variable indicating US federal holidays (*h*) and a continuous metric of daily average relative humidity (*RH*). Empty strata were removed from the dataset. The resulting regression equation for each dataset was:

$$E\left(N_{t,s}\right)=\exp\left\{\beta_{1}hw_{t,s}+\beta_{2}RH_{t,s}+\beta_{3}h_{t}\right\}$$

The estimated parameter of interest (*β*_1_) was used to calculate IRRs for heat exposure response under various dataset-outcome pairings. We performed sensitivity analyses under various definitions of exposure and within widened age strata (to ensure each stratum had more than 100 encounters total over the 4 years). (For sample estimation code, see eText 4.)

We assessed the similarity of IRRs estimated from each dataset (HCAI, OLDW), by encounter type (ED, inpatient), and outcome definition. The exposures and outcomes for both datasets are not independent. Accordingly, the standard approach of taking the difference of regression coefficients and utilizing independent standard errors to construct a confidence interval ^31,32^ would lead to either Type I or Type II errors depending on the covariance of the correlated coefficients (see explanation in eText 5). Another common method for judging significant difference between regression coefficients, i.e., non-overlapping 95% confidence intervals, has been shown to underestimate differences in regression coefficients.^33^ Owing to the limitations of these approaches, we combined aggregated versions of each dataset and used a dummy variable for dataset to directly capture the ratio of the two incidence rate ratios of interest. We present this ratio of incidence rate ratios (rIRR) in addition to graphical depictions of independently estimated IRRs. Our approach was informed by methods for seemingly unrelated regression,^34^ and a discussion of the interpretation of interaction terms in Poisson regression.^35^ We also were informed by studies investigating generalizability and incidence rate ratios: one that compared heart failure incidence rates across cohorts,^36^ and others that compared utilization rates of healthcare forms and vaccine safety during the early months of the COVID-19 pandemic in the US.^37,38^ We created a combined dataset of encounters, added the dataset label to the strata definition, introduced a dummy variable for dataset (*dt*), where *dt* = 0 if dataset = HCAI, and created interaction terms between this dummy variable and each other covariate (*dt* itself was not included as there were no strata containing data from both datasets by design):

$$E\left(N_{t,s}\right)=\exp\left\{\beta_{1}hw_{t,s}+\beta_{2}RH_{t,s}+\beta_{3}h_{t}+\beta_{4}\left[dt\times hw_{t,s}\right]+\beta_{5}\left[dt\times RH_{t,s}\right]+\beta_{6}\left[dt\times h_{t}\right]\right\}$$

The regression coefficient for the interaction term between the heatwave day and the dataset (*β*_4_) therefore represents the rIRR between the two datasets (see eText 6 for sample code showing the equivalence of this setup to the traditional unconditional Poisson approach in a case with fewer strata). We formatted the rIRR so that rIRR > 1 represents a stronger (i.e., more positive) association observed in the OLDW data, and rIRR < 1 represents a stronger association in the HCAI data. All analyses were performed using R version 4.2.1,^39^ and all conditional Poisson models were run using the gnm package.^40^

## Results

### Datasets

The total number of individuals in the OLDW dataset represented approximately 3.4% of the total in the California CDC dataset, with some key differences in terms of age and geographic distributions (Table 1). For example, the OLDW dataset included relatively more people than expected in Santa Clara County and relatively fewer people than expected in San Bernadino and Los Angeles counties. OLDW enrollees were present all 58 counties in California, with ED or inpatient encounters recorded in 56 counties. We observed minimal temporal trends by age-sex strata (eFig. 2) and county (eFig. 3); only the smallest strata had large shifts in total population over time. As expected, standardizing the OLDW dataset eliminated differences in terms of age, sex, county, or year versus the California CDC dataset.

### Incidence rates

Incidence rates of ED encounters for any cause and for heat, mental and/or behavioral disorders, cardiovascular disease, and respiratory disease were substantially higher for HCAI versus OLDW (Table 2). For example, rates of ED encounters for any cause were 335.8 encounters/million person–days in the crude OLDW dataset versus 827.3 encounters/million person–days in HCAI. Rates in OLDW were only slightly higher with versus without age–sex–county–year standardization, suggesting that differences in these factors did not explain the differences in rates observed between the crude OLDW and HCAI datasets. Only ED encounters for renal disease were of similar magnitude in the OLDW and HCAI datasets.

Rates of inpatient encounters for any cause, and for heat and mental/behavioral disorders were also higher in HCAI versus OLDW, while inpatient encounter rates for renal disease, cardiovascular disease, and respiratory disease were lower in the HCAI dataset. Standardization of the OLDW dataset had only a modest impact on all-cause or cause-specific inpatient incidence rates.

### Heat–health association comparisons

**Figure 1:**
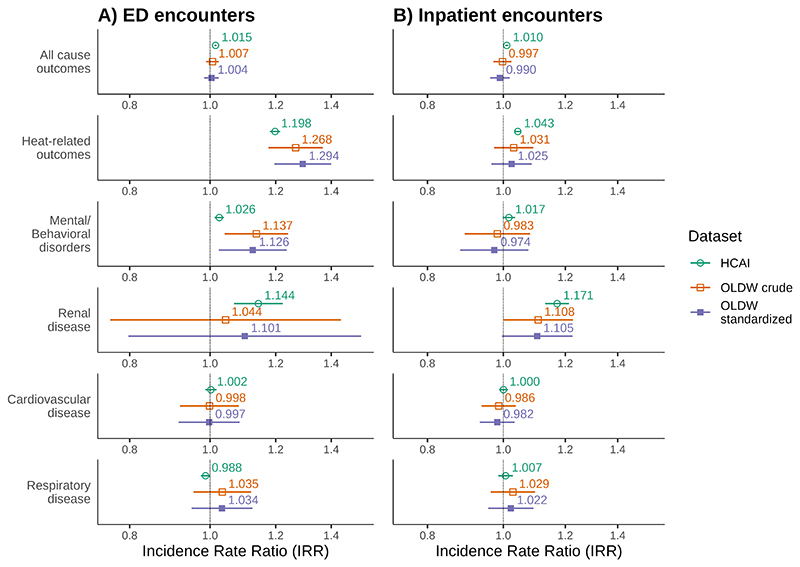
Incidence rate ratios (IRR) and 95% confidence intervals by outcome for (A) Emergency Department (ED) encounters and (B) Inpatient encounters on the any day of the heatwave compared to a non-heatwave day in the same county, year, month, and day of week. Datasets are California Department of Health Care Access and Information (HCAI) and crude and standardized Optum Labs Data Warehouse (OLDW). The x-axis is log-scaled in both panels.

Finally, we estimated the association between days of extreme heat and ED and inpatient encounters in each dataset (Fig. 1). Dispersion parameters for all but one model were below 1.5 indicating adequate control for over-dispersion (eTable 3). For ED encounters, the associations were typically in the same direction and broadly of similar magnitudes regardless of the dataset. For example, the association between heat wave day and all-cause ED encounters were IRRs of 1.015 in the HCAI dataset, 1.007 in the OLDW crude dataset, and 1.004 in the OLDW standardized dataset. For heat-related ED encounters, while all IRR were positive, OLDW IRRs were slightly higher, and with wider confidence intervals. This suggests that enrollees included in the OLDW dataset were marginally more likely than individuals in the HCAI dataset to experience a heat-related ED encounter on days of extreme heat. ED encounters for renal disease and mental and behavioral disorders followed a similar pattern: IRRs were positive in both datasets but marginally higher in the OLDW, and with wider confidence intervals. Calculated ratios of IRRs (rIRR) were aligned with the visual comparison of IRRs (Table 3 and Table 4). Specifically, most crude and standardized rIRR were similar across outcomes, although mental health and heat-related rIRRs indicated marginally stronger associations in OLDW data than in HCAI. Both mental health and heat-related rIRR indicated stronger associations in OLDW data than in HCAI. IRRs for inpatient encounters were similar to those for ED encounters, but with consistently wider confidence intervals in OLDW (Fig. 1B).

There was some evidence of effect measure modification by age-group within each dataset (eFig. 4 and 5). For mental health related ED encounters, IRR in HCAI remained relatively similar across age-groups, but the OLDW IRR was higher for adults aged 18-64 than adults aged 65 and older. This may suggest that some OLDW patients (perhaps preferentially high-risk patients) left the OLDW system when they became eligible for coverage under Medicare. For inpatient encounters, the strongest effect-modification by age was seen in HCAI for renal disease; IRR for adults aged 18-64 was much lower than for adults aged 65 and older.

Similarly, we observed some minor differences in IRR for each dataset depending on heat-wave definition (eFig. 6 and 7). IRRs for all-cause, heat-related, and mental health-related ED encounters were similar regardless of heat-wave definition, but the IRR for renal disease in OLDW (but not HCAI) were highest on the 2nd+ day of heatwaves. For inpatients, the IRR for all-cause, heat-related and renal disease conditions were similar regardless of heatwave definition, but the relative rate of mental health-related outcomes in OLDW (but not HCAI) were lower on the first day of a heatwave.

## Discussion

The substantial health dangers posed by days of extreme heat are well documented. Recent heat–health studies have leveraged OLDW owing to its large spatial and temporal span and the wide age range of individuals covered.^11–15^ But being enrolled in a commercial health insurance plan may itself serve as a marker of lower risk of injury due to climate hazards, potentially limiting the generalizability of results derived from OLDW and similar databases of insured individuals. This paper addresses the question of generalizability of heat–health associations derived from individuals with commercial health insurance, specifically individuals with commercial or Medicare Advantage health insurance enrolled in OLDW in California. For ED encounters, we found that the associations between extreme heat and health were largely consistent in direction and magnitude across datasets. Specifically, the incidence rate ratios for heat-related ED encounters and mental-health ED encounters were positive in both datasets, albeit with a marginally larger magnitude and wider confidence intervals in OLDW. For inpatient encounters, IRRs were similar between the two datasets, but rate ratio estimates for OLDW had wider confidence intervals. Thus, if results were only available from the OLDW among enrollees in California, some associations between extreme heat and health harm may be missed. While the OLDW dataset may differ from the HCAI dataset in important ways, the direction and magnitude of the associations on the ratio scale between extreme heat and ED and inpatient encounters in OLDW are broadly consistent with those found in the HCAI dataset. Beyond issues of statistical precision, the similarity of IRRs for key health outcomes supports the use of OLDW data in quantifying the effect of extreme heat on human health, especially for ED encounters.

These results are contextualized by an interrogation of dataset composition and underlying factors that drive healthcare utilization of the individuals in each dataset. The age and geographic distribution of the OLDW and HCAI datasets differ appreciably, with the crude OLDW data skewing younger and preferentially located in Santa Clara, San Diego, Orange, and Contra Costa Counties. Moreover, average incidence rates of ED and inpatient encounters were also substantially higher in the HCAI versus OLDW dataset, suggesting that the individuals in the OLDW dataset tended to be healthier than the HCAI dataset. However, standardizing the OLDW dataset to match the state population in terms of age, sex, county, and year had minimal impact on average incidence rates, suggesting that these differences do not appreciably contribute to the observed differences in incidence rates. The HCAI dataset was much larger, reflecting the ED and inpatient encounters of approximately 29 million residents of California. In comparison, the California population of OLDW reflects the medical claims of just under 1 million California residents, or about 3.4% of the state’s population during this time. As a result, estimated incidence rate ratios in the OLDW dataset have a larger variance (i.e., wider confidence intervals) versus those derived from the HCAI dataset, and higher probability of missing some important associations of small magnitude.

Differences in incidence rates also emphasize a central question of this paper – do the factors that lead to these differences affect the ability to use OLDW data to characterize changes in population-level health resulting from extreme heat? In the absence of a comprehensive population-based dataset that includes insurance status and type, we can address this question through discussion of the unmeasured individual factors that may lead to differences in ED and inpatient encounter rates by insurance status. Individuals without health insurance may on average have high healthcare utilization due to greater vulnerability, susceptibility and other factors.^41–43^ Conversely, large systematic reviews have shown that having insurance is associated with both better health status and higher levels of healthcare utilization.^16^ Relevant to healthcare utilization studies in California, a state with approximately 2 million undocumented individuals at high risk for adverse health effects,^44^ undocumented status was not associated with higher healthcare utilization^45^ or ED encounter rates compared to insured populations.^46^ Research findings on the relationship between insurance status and inpatient encounters have shown that uninsured patients have shorter length of stay compared to publicly insured patients,^47^ and higher odds of discharge or transfer as compared to privately insured patients.^48^ However research findings on the relationship between insurance status and ED encounters rates are mixed: recent studies have reported higher rates of ED encounters among both the insured^42^ and uninsured.^49,50^ Specific to climate hazards, uninsured individuals may be at greater risk of suffering the worst impacts and thus can have higher utilization during periods of extreme weather.^16,17^ A patient’s insurance status has also been associated with differential assignment of diagnosis codes by providers (perhaps given implications for billing),^51–53^ which could impact results of studies in which outcomes are identified using diagnosis codes. Considering these factors, the likelihood of a trip to the emergency room (and any resulting diagnosis) is a function of financial status, underlying health conditions, access to healthcare facilities and other community resources, and individual behavior and preferences, all of which are not neatly stratified by insurance status alone.

Given the various determinants of healthcare utilization among the insured and insured, and our present results, we believe that OLDW can continue to be a resource for understanding the impacts of environmental exposures on human health. In both datasets, several key markers of the health impacts of extreme heat, including ED encounters for heat-related health, mental health, and renal disease, were in the same direction and had similar magnitudes. Although some of these associations were marginally stronger in OLDW than in HCAI, ultimately key outcomes were similarly and positively identified. The OLDW (and HCAI) results in this study align with others in California that characterized the relationship between extreme heat and human health, with the strongest associations seen for rates of mental health outcomes, renal disease, and dehydration/heat illness.^54–57^ As well, the OLDW is a unique resource in that it contains individuals of all ages with wide spatial and temporal spans in the contiguous US. A similar analysis at the national scale characterized the association between heat and ED encounters for a variety of causes.^15^

We have several recommendations for others using OLDW data. Whenever possible, OLDW results should be contextualized using a population-based dataset, even for a geographic subset of the total dataset. Second, population standardization can be considered but may not be necessary in future analysis, owing to the many factors that drive differences in ED and inpatient encounter rates that cannot be standardized given the available data. Finally, a strength of the OLDW is its coverage of individuals of all ages, across time and space in the contiguous US, thus ideal research questions will leverage the availability of data across these dimensions.

Finally, our results should be interpreted considering the following limitations. First, we did not have access to many individual-level variables in OLDW that would be useful in stratifying results, e.g., income or race/ethnicity. As such, we cannot demonstrate the degree to which the OLDW California population differs from that of California on these dimensions, nor can we standardize estimates accordingly. Second, the HCAI data lacked information on the expected payer, limiting our ability to directly compare the effects of heat among the publicly insured, privately insured, and uninsured. Third, neither dataset included information on people that died during or prior to admission, potentially systematically excluding the small fraction of individuals most severely impacted on days of extreme heat. Fourth, our comparisons were performed for OLDW enrollees and residents in California, and the extent of generalizability may vary in other US states.

## Conclusions

In this study of the impact of extreme heat on healthcare utilization, we compared incidence rate ratios (IRR) for emergency depart (ED) and in-patient hospitalization encounters among adult residents of California included in a dataset of commercially insured individuals (OLDW) and a dataset representing essentially all individuals in California (HCAI). Rate ratios for days extreme heat and ED encounters were largely of similar magnitudes and directions despite differences in baseline incidence rates. Rate ratios for days of extreme heat and inpatient encounters in OLDW and HCAI had overlapping confidence intervals, but confidence intervals for OLDW were much wider. Population standardization did not meaningfully change the ratio of IRRs between these two populations. Despite some important limitations, a dataset of commercially insured individuals (OLDW), appears to be appropriate for use in quantifying the effect of extreme heat on rates of related ED encounters, and to a lesser extent, inpatient encounters.

## Supplementary Material

Supplemental Digital Content

## Figures and Tables

**Table 1:** Annual average population demographics and between-dataset ratios, averaged across years.

	OLDW enrollees in CA	CDC population data for CA	Ratio
**Total population**	1,005,765	31,347,773	0.03
% Female	50.1	50.6	0.99
*Age (year) categories:*			
% in 18 to 24	11.3	17.3	0.66
% in 25 to 34	20.5	18.6	1.10
% in 35 to 44	20.8	16.5	1.26
% in 45 to 54	19.0	16.4	1.16
% in 55 to 64	14.7	14.6	1.01
% in 65 to 74	7.5	9.5	0.79
% in 75+	6.1	7.1	0.86
*County a:*			
% in Los Angeles (06037)	18.7	26.1	0.72
% in San Diego (06073)	11.4	8.5	1.34
% in Orange (06059)	9.4	8.2	1.15
% in Riverside (06065)	4.6	5.9	0.77
% in San Bernardino (06071)	3.6	5.3	0.69
% in Santa Clara (06085)	10.7	4.9	2.18
% in Alameda (06001)	5.9	4.3	1.38
% in Sacramento (06067)	3.3	3.8	0.85
% in Contra Costa (06013)	3.6	2.9	1.26
% in Other	28.7	30.1	0.95

CA indicates California; CDC, Centers for Disease Control and Prevention; OLDW, Optum Labs Data Warehouse

a Large counties are defined by CDC population > 1 million.

**Table 2:** Incidence rates (encounters / 1M person–days) by outcome

	ED encounters				Inpatient encounters		
Outcome	OLDW crude	OLDW standardized	HCAI		OLDW crude	OLDW standardized	HCAI
All-cause	335.8	362.7	827.3		153.8	163.4	276.3
Heat-related	16.4	17.9	30.4		30.1	34.6	67.1
Renal disease	0.8	1.0	0.9		9.0	10.9	4.3
Mental / Behavioral disorders	12.9	13.9	40.5		11.7	12.9	19.3
Cardiovascular disease	16.1	17.6	25.9		41.1	47.7	37.8
Respiratory disease	17.3	19.5	44.3		24.3	28.1	14.8

ED indicates Emergency Department; HCAI, California Department of Health Care Access and Information; OLDW, Optum Labs Data Warehouse

**Table 3:** Coefficients and confidence intervals of the conditional quasi-Poisson model by outcome for ED and Inpatient encounters, using the HCAI dataset and standardized OLDW dataset.

	ED encounters			Inpatient encounters	
Outcome	rIRR	95%CI		rIRR	95%CI
All-cause	0.989	(0.969, 1.009)		0.980	(0.953, 1.008)
Heat-related	1.080	(0.999, 1.168)		0.982	(0.927, 1.040)
Renal disease	0.963	(0.718, 1.292)		0.943	(0.850, 1.047)
Mental / Behavioral disorders	1.098	(1.004, 1.201)		0.958	(0.872, 1.052)
Cardiovascular disease	0.995	(0.917, 1.080)		0.982	(0.933, 1.033)
Respiratory disease	1.047	(0.964, 1.137)		1.016	(0.950, 1.086)

ED indicates Emergency Department; HCAI, California Department of Health Care Access and Information; OLDW, Optum Labs Data Warehouse

**Note:** rIRR > 1 represents a stronger association observed in the OLDW data, and rIRR < 1 represents a stronger association in the HCAI data

**Table 4:** Coefficients and confidence intervals of the conditional quasi-Poisson model by outcome for ED and Inpatient encounters, using the HCAI dataset and crude OLDW dataset.

	ED encounters			Inpatient encounters	
Outcome	rIRR	95%CI		rIRR	95%CI
All-cause	0.992	(0.973, 1.011)		1.014	(0.987, 1.042)
Heat-related	1.059	(0.977, 1.148)		1.030	(0.971, 1.092)
Renal disease	0.913	(0.657, 1.269)		1.055	(0.945, 1.176)
Mental / Behavioral disorders	1.108	(1.012, 1.214)		1.049	(0.952, 1.156)
Cardiovascular disease	0.996	(0.916, 1.083)		1.026	(0.974, 1.081)
Respiratory disease	1.048	(0.963, 1.140)		1.035	(0.966, 1.109)

ED indicates Emergency Department; HCAI, California Department of Health Care Access and Information; OLDW, Optum Labs Data Warehouse

**Note:** rIRR > 1 represents a stronger association observed in the OLDW data, and rIRR < 1 represents a stronger association in the HCAI data
