# Supplementary material for: Generalizability of heat-related health risk associations observed in a large healthcare claims database of patients with commercial health insurance
Source: Epidemiology. Author manuscript; Available in PMC 2024 Sep 20. (PMC7616519; doi:10.1097/EDE.0000000000001781)
Supplement: Supplemental Digital Content [file EMS197742-supplement-Supplemental_Digital_Content.docx]

# Supplemental Information

eText 1: Limitations on linking datasets

eText 2: Function for exposure definition

eText 3: Sample calculation for case fraction weighting

eText 4: GNM R code defining the outcome

eText 5: Explanation of inferential error using the standard test for regression coefficient difference

eText 6: Demonstration of conditional versus unconditional Poisson models in calculating rIRR

eTable 1. International Classification of Diseases 9^th^ and 10^th^ revision codes used to define outcomes

eTable 2. Heatwave days in each year. Total county summer-time days in each year is 8,874 (58 counties × 153 summer days, i.e., May 1 to Sept 30).

eTable 3. Dispersion parameters from conditional quasi-Poisson modeling by model type

eFigure 1. Process for how OLDW claims are assigned as Inpatient (IP), Long-term, or ED claims. Only ED and IP were used in this study

eFigure 2. Comparison of temporal trend of scaled populations by age-sex strata in the CDC race-bridge datasets. Facet labels are the joined age-sex strata and are ordered by the size of the population in each group

eFigure 3. Comparison of temporal trends in county-specific scaled OLDW and CDC population. Facet labels are the county FIPS id and are ordered by the size of the population in each group (from lowest in the top left, to highest in the bottom right).

eFigure 4. ED incidence rate ratios for any heatwave day by age group sensitivity (40 year age groups). Yaxis has a log scale. The ‘All ages’ category is what is plotted in Figure 1, panel A.

eFigure 5. IP incidence rate ratios for any heatwave day by age group sensitivity (40 year age groups). Yaxis has a log scale. The ‘All ages’ category is what is plotted in Figure 1, panel B.

eFigure 6. ED incidence rate ratios for all ages by heatwave definition. Yaxis has a log scale. The ‘Any day of a heatwave’ category is what is plotted in Figure 1, panel A.

eFigure 7. IP incidence rate ratios for all ages by heatwave definition. Yaxis has a log scale. The ‘Any day of a heatwave’ category is what is plotted in Figure 1, panel B.

## eText 1: Limitations on linking datasets

It is likely that HCAI records contain all or most OLDW events, but we cannot verify this without linking the datasets, which we were restricted from doing. In addition to differences in utilization and vulnerability, other administrative and data processing factors may contribute to differences in populations and heat-health associations in these datasets. Some OLDW records may not be included in HCAI on a systematic basis (e.g., claims from centers that are not required to report to HCAI), or on a random basis (one-off omissions in hospital reporting to HCAI that would occur within allowable reporting error thresholds). In addition, in the HCAI, if ED visits or hospitalizations resulted in mortalities, these encounters may be grouped under encounters in a mortality dataset, which this study did not have access to. The method used to define ED visits is another source of differences between the datasets. In HCAI, ED visits and hospitalization datasets are provided separately by HCAI, and did not include revenue codes or other identifying information for specific encounters. This process likely varies on a facility-by-facility basis. In OLDW, ED visits are identified from claims by a set of characteristics that includes AMA codes, revenue codes, and CPT codes. Even for the same encounter, the specific claim or record fields may differ between the two datasets, owing to preservation of anonymity or other processing steps. These field differences include participant age, sex and home zipcode, claim service date and ICD-10 code order. Location of the reporting facility is not included in the OLDW dataset (by design, to preserve anonymity) or HCAI (owning to limitations in resources of this study). These sources of difference between two large administrative datasets would be almost impossible to completely remove, and reflect a series of processing and administrative decisions over which the research team had little or no control. Instead, understanding these and other complexities informs the discussion of heat-health associations derived from the two datasets and give guidance to future analyses and data requests.

## eText 2: Function for exposure definition

# Function to define a heatwave exposure, being a sequence of 2 or more days greater than the # Xth percentile. Inputs are a binary vector indicating whether a day is in the Xth percentile

# and an integer indicating the minimum number of consecutive days to count as a heatwave

get_heatwave_days <- function(IS_X, ndays) {

# Replace missing values with FALSE

IS_X[is.na(IS_X)] <- FALSE

# Compute the run-length encoding of IS_X

rlepX <- rle(IS_X)

# Compute the indices of the start and end of each run

end <- cumsum(rlepX$lengths)

start <- c(1, lag(end)[-1] + 1)

# Create a data frame with the start and end indices, the value of each run, and its length

hw_days <- data.frame(start, end, val = rlepX$values, len = rlepX$lengths) %>%

# Filter runs that are in the Xth percentile and are longer than ndays

filter(val == TRUE & len >= ndays)

# Initialize a vector to store the heatwave numbers

HEATWAVE <- rep(NA, length = length(IS_X))

# If there are any heatwaves

if(nrow(hw_days) > 0) {

# Loop over each heatwave

for(j in 1:nrow(hw_days)) {

st <- hw_days$start[j] # Start index of the heatwave

ed <- hw_days$end[j] # End index of the heatwave

len <- hw_days$len[j] # Length of the heatwave

# Assign the heatwave number to the corresponding indices in the HEATWAVE vector

HEATWAVE[st:ed] <- rep(j, length = len)

}

}

# Return a vector of integers indicating the heatwave number for each day,

# or NA if the day is not part of a heatwave

return(HEATWAVE)

}

# Generate a binary vector of temperatures

set.seed(123)

temps <- rnorm(30, mean = 80, sd = 5)

IS_X <- temps > quantile(temps, 0.975)

# Get the number of days in a heatwave with a minimum length of 2 days

hw_days <- get_heatwave_days(IS_X, 2)

# Print the results

print(hw_days)

## eText 3: Sample calculation for case fraction weighting

Following conventional methods, we standardized daily counts of events (ED visits and IP hospitalizations) observed in the OLDW using weights that reflected CDC population distributions by strata within each year:

$f_{i,CDC}=\frac{Population in CDC strata i}{Total CDC population in year Y}$ (1)

$f_{i,OLDW}=\frac{Population in OLDW strata i}{Total OLDW population in year Y}$ (2)

$N_{i, std,decimal}=N_{i}*\frac{f_{i, CDC}}{f_{i,OLDW}}$ (3)

where $N_{i}$ is raw encounter numbers and $N_{i,std,decimal}$ represents the standardized encounter numbers in age-sex-county-year strata *i* of the OLDW population, a decimal value. We did not have information on which HCAI encounters were also in the OLDW dataset, so we were unable to calculate stabilized weights. To prepare$N_{i,std,decimal}$ for Poisson modeling and to reduce count inflation due to rounding, we used the following equations to minimally adjust decimal counts into integers:

$p_{i}= mod(N_{i, std,float} , 1)$ (4)

$C_{i}=\Pr\left( X=1 \right) \mathrm{where} C \sim Bernoulli(p_{i})$ (5)

$N_{i,std,int}=floor \left( N_{i,std,decimal} \right)+C_{i}$ (6)

where $p_{i}$ is the decimal part of $N_{i,std,decimal}$ (found by taking the modulus of $N_{i,std,float}$by 1), $C_{i}$ is an integer {0, 1} from a Bernoulli trial with probability $p_{i}$, and $N_{i,std,int}$ is the resulting integer value for standardized case counts. See the below for an example of this calculation.

## eText 4: GNM R code defining the outcome.

library(gnm) # for conditional and quasi-Poisson

str(case_data)

# has columns:

# STAY_START: date, the day the encounter starts

# DAY_OF_WEEK: string, for day of week

# MONTH: string, for month

# YEAR: string, for year

# STATE_COUNTY_FIPS: string, the 5 digit state-county FIPS code

# AGEGRP: string, age group category

# N_ENCOUNTERS: integer, the number of encounters that start on this day

# PERSON_TIME_AT_RISK: integer, the amount of people at risk on that day

str(weather_data)

# has columns:

# DATE: date, the day

# IS_HEATWAVE: logical, true if heatwave day (any, 1^st^ day, 2^nd^+ day)

# RH_MEAN: numeric, the mean relative humidity (RH) that day

# IS_HOLIDAY: logical, true if is US holiday

# join on case_data$STAY_START, weather_data$DATE

case_data <- left_join(case_data, weather_data, by = <insert join code>)

# define the stratum

# for each county, by day of week, in a particular month and year

stratum_cols <- c('STATE_COUNTY_FIPS', 'DAY_OF_WEEK', 'MONTH', 'YEAR')

case_data$stratum <- interaction(case_data[, stratum_cols])

# Create the offset because the number of people at risk changes over time for

# Because of multinomial distribution take the log

case_data$pop_offset <- log(case_data$person_time_at_risk)

# run the model, control for RH and HOLIDAY, and condition on strata

# offset by the log of the population at risk

mod_std <- gnm(N_ENCOUNTERS ~ IS_HEATWAVE + RH_MEAN + IS_HOLIDAY,

data = case_data,

family = quasipoisson,

offset = pop_offset,

eliminate = factor(stratum))

# get model coefficients

summary(mod_std)

## eText 5: Explanation of inferential error using the standard test for regression coefficient difference.

This is a simple representation of the study design of our paper. Given two simple linear models, regress the same exposure $X$ against an outcome $Y$ in two populations, so $Y_{1}$ and $Y_{2}$:

$$Y_{1}=\beta_{1}X+ \varepsilon_{1}$$

$$Y_{2}=\beta_{2}X+ \varepsilon_{2}$$

Using the MLE of $\beta$, i.e., $\hat{\beta}=\left( X^{'}X \right)^{-1}X'Y$, we can derive the formulae for $\hat{\beta}_{1}$and$\hat{\beta}_{2}$:

$$\hat{\beta}_{1}=Cov(Y_{1},X)/Var(X)$$

$$\hat{\beta}_{2}=Cov(Y_{2},X)/Var(X)$$

We are interested in statistically testing if $\hat{\beta}_{1}$ and $\hat{\beta}_{2}$ are different in the case where $\hat{\beta}_{1}$ and $\hat{\beta}_{2}$ are correlated, given that they are both functions of $Var(X)$_._ The steps below outline why we cannot use the standard test of difference of regression coefficients (Altman 2003^[[1]](#footnote-2)^) given the above study design.

First, construct an estimator of the difference of $\hat{\beta}_{1}$ and $\hat{\beta}_{2}$:

$$\hat{\theta}=\hat{\beta}_{2}-\hat{\beta}_{1}$$

The variance of this estimator follows from the basic property of the variance $Var\left( A+B \right)= Var\left( A \right)+Var\left( B \right)+2 Cov(A,B)$. If $A=\hat{\beta}_{1}$ and $B=-\hat{\beta}_{2}$ then:

$$Var\left( \hat{\theta} \right)= Var\left( \hat{\beta}_{1} \right)+Var\left( \hat{\beta}_{2} \right)-2 Cov(\hat{\beta}_{1},\hat{\beta}_{2})$$

The null hypothesis is: $H_{0}: \theta=0$

In the standard test of the difference of regression coefficients (Altman 2003), it is assumed that $\hat{\beta}_{1}$ and $\hat{\beta}_{2}$ are not correlated, hence $Cov\left( \hat{\beta}_{1},\hat{\beta}_{2} \right)=0$, which leads to $Var\left( \hat{\theta} \right)=Var\left( \hat{\beta}_{1} \right)+Var\left( \hat{\beta}_{2} \right)$.

However, this does not hold in our case. $Cov\left( \hat{\beta}_{1},\hat{\beta}_{2} \right) \neq0$ because $\hat{\beta}_{1}$ and $\hat{\beta}_{2}$are both functions of $Var(X)$. So if the standard test is used, i.e., so $Var\left( \hat{\theta} \right)= Var\left( \hat{\beta}_{1} \right)+Var\left( \hat{\beta}_{2} \right)$, this would lead to one of two cases of inferential error, depending on the sign of $Cov\left( \hat{\beta}_{1},\hat{\beta}_{2} \right)$.

If $Cov\left( \hat{\beta}_{1},\hat{\beta}_{2} \right)$ > 0, the standard test will over-estimate $Var\left( \hat{\theta} \right)$ (i.e., the true variance will be smaller than as predicted by the standard test). This will lead to Type II errors, failing to reject the $H_{0}$ when $\theta\neq0$, because of the larger range of $Var\left( \hat{\theta} \right)$ using the standard test.

If $Cov\left( \hat{\beta}_{1},\hat{\beta}_{2} \right)$ < 0, the standard test will under-estimate $Var\left( \hat{\theta} \right)$ (i.e., the true variance will be greater than as predicted by the standard test). This will lead to Type I errors, rejecting $H_{0}$ when $\theta=0$, because of the smaller range of $Var\left( \hat{\theta} \right)$ using the standard test.

The solution to accounting for the $Cov\left( \hat{\beta}_{1},\hat{\beta}_{2} \right)$ is to use a model that contains these terms together (e.g., through SUR as we chose) or via bootstrapping.

*eText 6: Demonstration of conditional versus unconditional Poisson models in calculating rIRR*

*
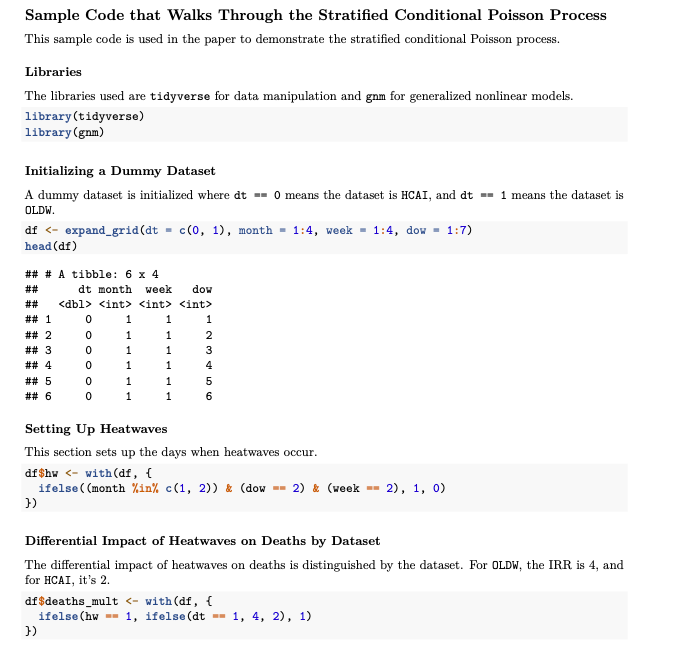
*


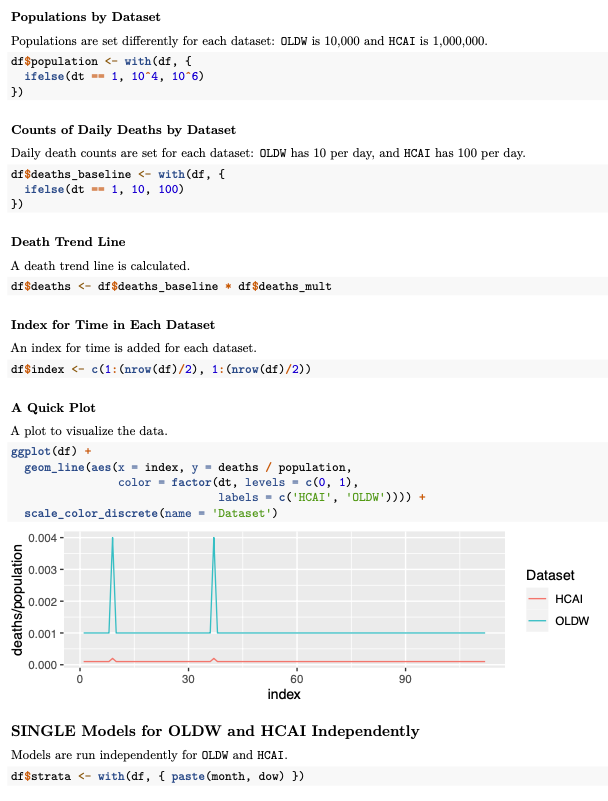


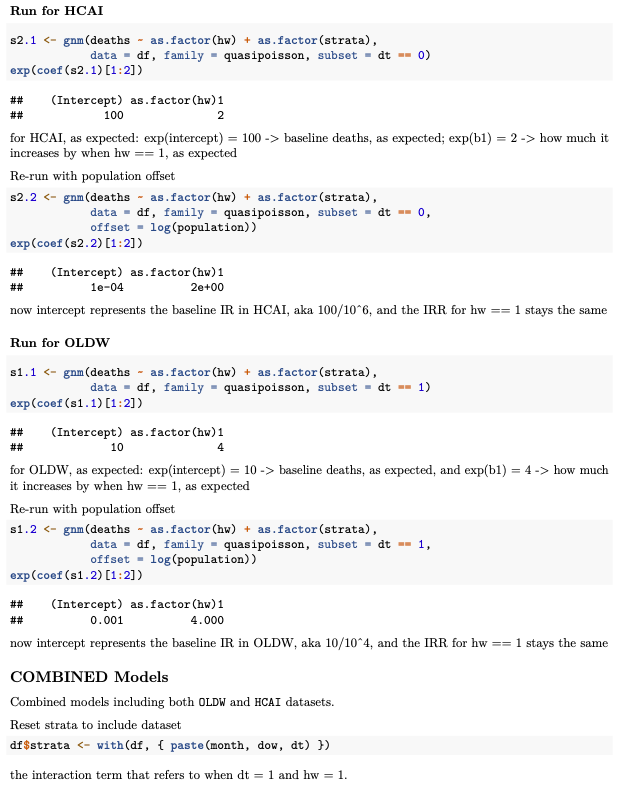


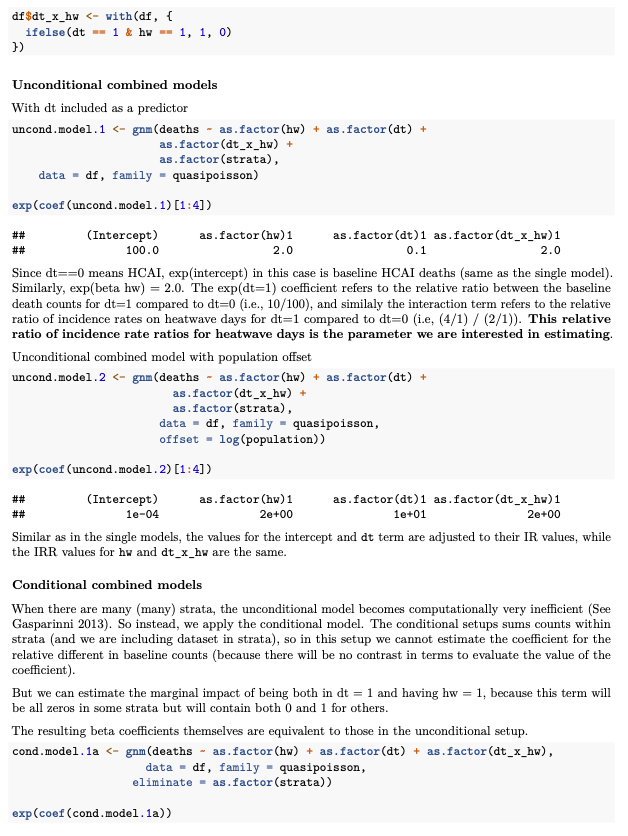
 ^[[2]](#footnote-3)^


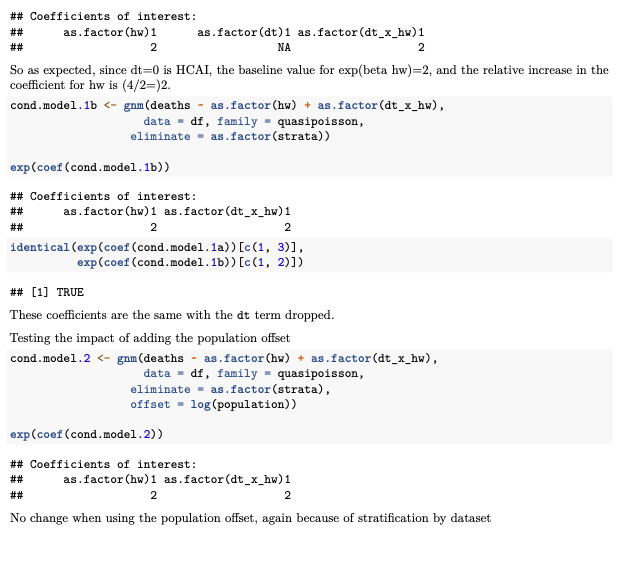


## Tables

**eTable 1.** *International Classification of Diseases 10th revision* (ICD-10) codes used to define outcomes.

| **Outcome** | **ICD-9 Codes** | **ICD-10 Codes** |
| --- | --- | --- |
| All-cause | Any recorded claim | Any recorded claim |
| Heat-related | 992, 276, E9000, E9009 Excluded: E9001 | T67, X30, X32, E86, E87 in any diagnosis code position  Excluded: W92 in any diagnosis code position |
| Renal disease | 580 through 589  in the 1^st^ diagnosis code position | N00, N01, N02, N03, N04, N05, N08, N17, N18, N19, N25, N26, N27  in the 1^st^ diagnosis code position |
| Mental / Behavioral disorders | 290 through 319 in the 1^st^ diagnosis code position | “F” as the first character of the 1^st^ diagnosis code position |
| Cardiovascular disease | 390 through 459 in the 1^st^ diagnosis code position | “I” as the first character of the 1^st^ diagnosis code position |
| Respiratory disease | 460 through 519 in the 1^st^ diagnosis code position | “J” as the first character of the 1^st^ diagnosis code position |

**eTable 2**. Heatwave days in each year. Total county summer-time days in each year is 8,874 (58 counties × 153 summer days, i.e., May 1 to Sept 30).

| **Year** | **N county-days**  **> 97.5^th^ percentile** | **% of total** | **N county-days**  **in a heatwave** | **% of total** |
| --- | --- | --- | --- | --- |
| 2012 | 407 | 4.6 | 381 | 4.3 |
| 2013 | 506 | 5.7 | 474 | 5.3 |
| 2014 | 485 | 5.5 | 449 | 5.1 |
| 2015 | 651 | 7.3 | 595 | 6.7 |
| 2016 | 597 | 6.7 | 549 | 6.2 |
| 2017 | 981 | 11.1 | 963 | 10.9 |
| 2018 | 436 | 4.9 | 410 | 4.6 |
| 2019 | 384 | 4.3 | 358 | 4.0 |

**eTable 3**. Dispersion parameters from conditional quasi-Poisson modeling by model type.

| **Standardized** | **Outcome** | **Population** | **ED visit dispersion parameter** | **Hospitalization dispersion parameter** |
| --- | --- | --- | --- | --- |
| Standardized | All cause | OLDW | 1.550 | 1.380 |
|  | All cause | HCAI | 1.370 | 1.030 |
|  | All cause | Combined | 1.490 | 1.250 |
|  | Heat-related | OLDW | 0.999 | 1.010 |
|  | Heat-related | HCAI | 1.370 | 1.030 |
|  | Heat-related | Combined | 1.130 | 1.020 |
|  | Renal disease | OLDW | 1.340 | 1.300 |
|  | Renal disease | HCAI | 1.230 | 1.040 |
|  | Renal disease | Combined | 1.280 | 1.170 |
|  | Mental/Behavioral | OLDW | 1.020 | 1.000 |
|  | Mental/Behavioral | HCAI | 1.230 | 1.040 |
|  | Mental/Behavioral | Combined | 1.130 | 1.020 |
|  | Cardiovascular | OLDW | 1.360 | 1.300 |
|  | Cardiovascular | HCAI | 1.010 | 1.020 |
|  | Cardiovascular | Combined | 1.060 | 1.140 |
|  | Respiratory | OLDW | 0.996 | 0.984 |
|  | Respiratory | HCAI | 1.010 | 1.020 |
|  | Respiratory | Combined | 1.010 | 1.010 |
| Not standardized | All cause | OLDW | 1.340 | 1.330 |
|  | All cause | HCAI | 1.060 | 0.964 |
|  | All cause | Combined | 1.180 | 1.120 |
|  | Heat-related | OLDW | 0.989 | 0.999 |
|  | Heat-related | HCAI | 1.060 | 0.964 |
|  | Heat-related | Combined | 1.030 | 0.979 |
|  | Renal disease | OLDW | 1.290 | 1.280 |
|  | Renal disease | HCAI | 1.030 | 1.020 |
|  | Renal disease | Combined | 1.150 | 1.160 |
|  | Mental/Behavioral | OLDW | 1.000 | 0.996 |
|  | Mental/Behavioral | HCAI | 1.030 | 1.020 |
|  | Mental/Behavioral | Combined | 1.020 | 1.010 |
|  | Cardiovascular | OLDW | 1.390 | 1.300 |
|  | Cardiovascular | HCAI | 1.210 | 1.020 |
|  | Cardiovascular | Combined | 1.300 | 1.160 |
|  | Respiratory | OLDW | 1.010 | 1.000 |
|  | Respiratory | HCAI | 1.210 | 1.020 |
|  | Respiratory | Combined | 1.110 | 1.010 |

## Figures

**
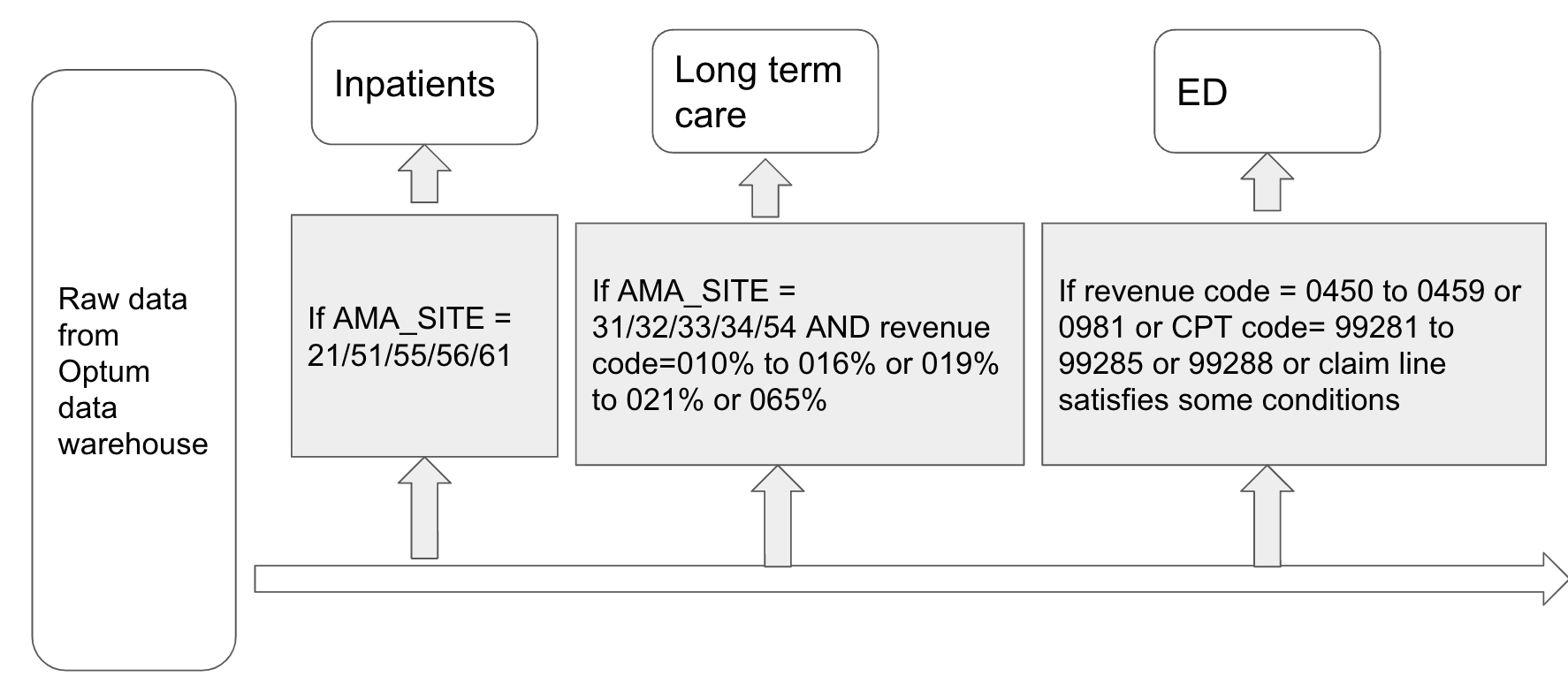
**

**eFigure 1.** Process for how OLDW claims are assigned as Inpatient (IP), Long-term, or ED claims. Only ED and IP were used in this study.


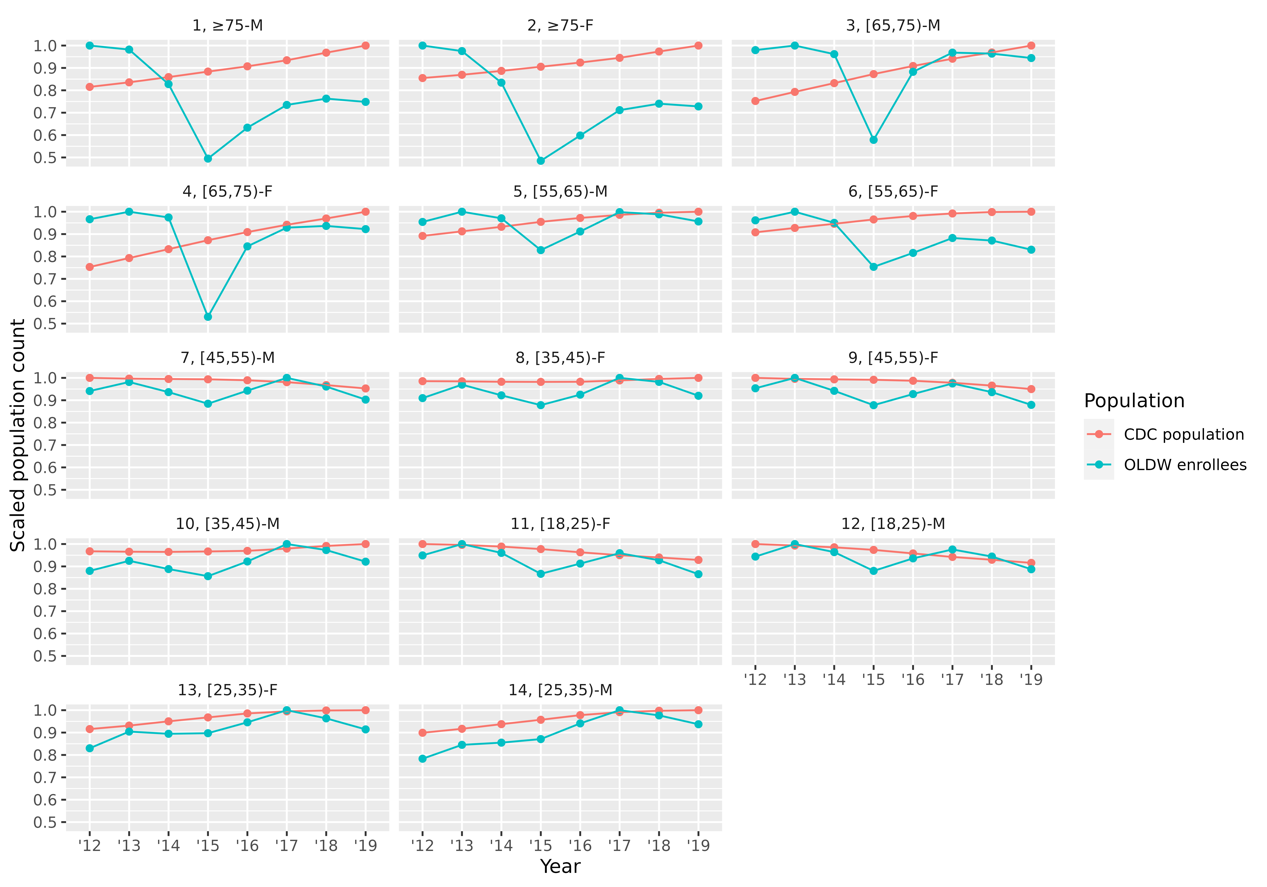


**eFigure 2.** Comparison of temporal trend of population counts by age-sex strata. Within each strata, the population counts are scaled by the maximum annual population count in each group to facilitate comparison of trends across populations. Facets are ordered by the size of the population in each group (from lowest in the top left, to highest in the bottom right).


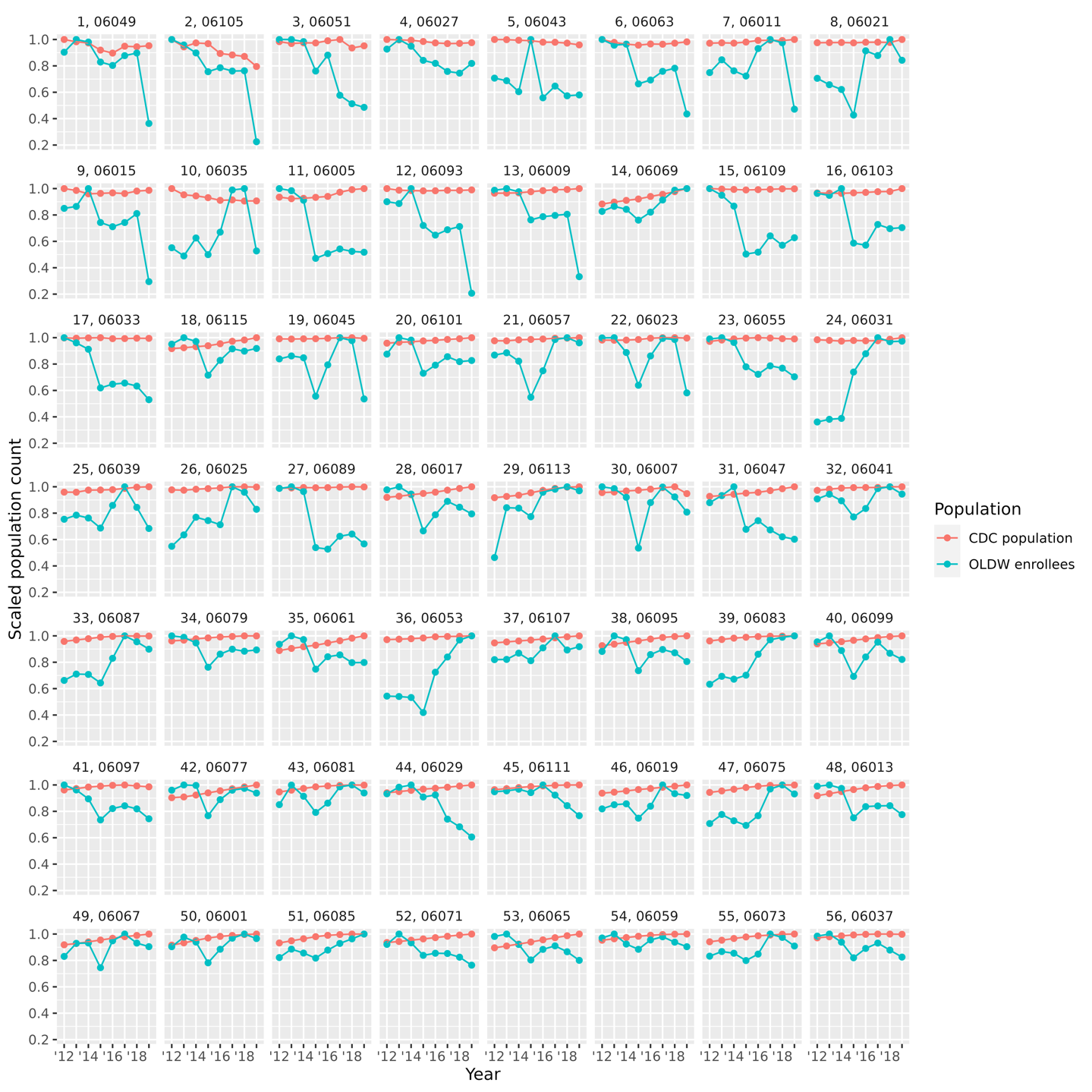


**eFigure 3.** Comparison of temporal trends in county-specific scaled OLDW and CDC population. Facet labels are the county FIPS id and are ordered by the size of the population in each group (from lowest in the top left, to highest in the bottom right).


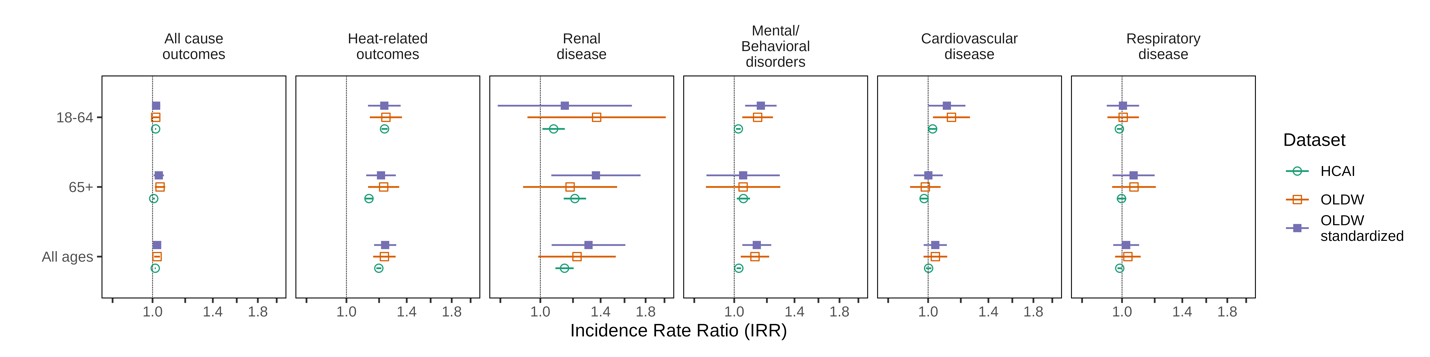


**eFigure 4**. ED incidence rate ratios for any heatwave day by age group sensitivity (40 year age groups). Yaxis has a log scale. The ‘All ages’ category is what is plotted in Figure 1, panel A.


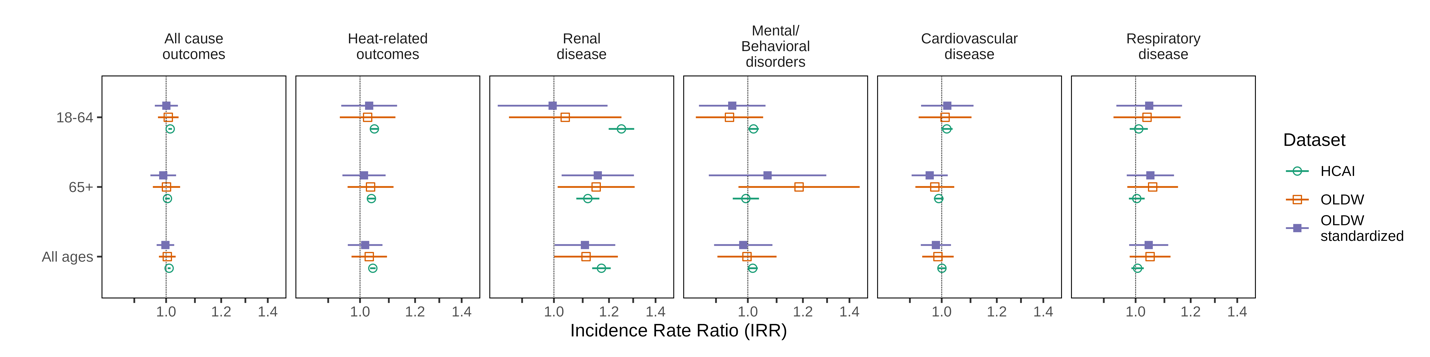


**eFigure 5**. IP incidence rate ratios for any heatwave day by age group sensitivity (40 year age groups). Yaxis has a log scale. The ‘All ages’ category is what is plotted in Figure 1, panel B.


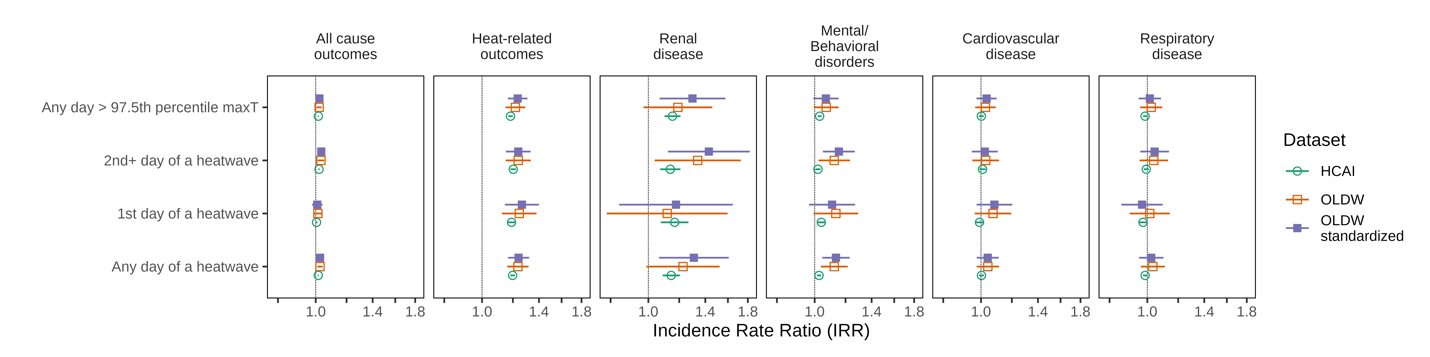


**eFigure 6**. ED incidence rate ratios for all ages by heatwave definition. Yaxis has a log scale. The ‘Any day of a heatwave’ category is what is plotted in Figure 1, panel A.


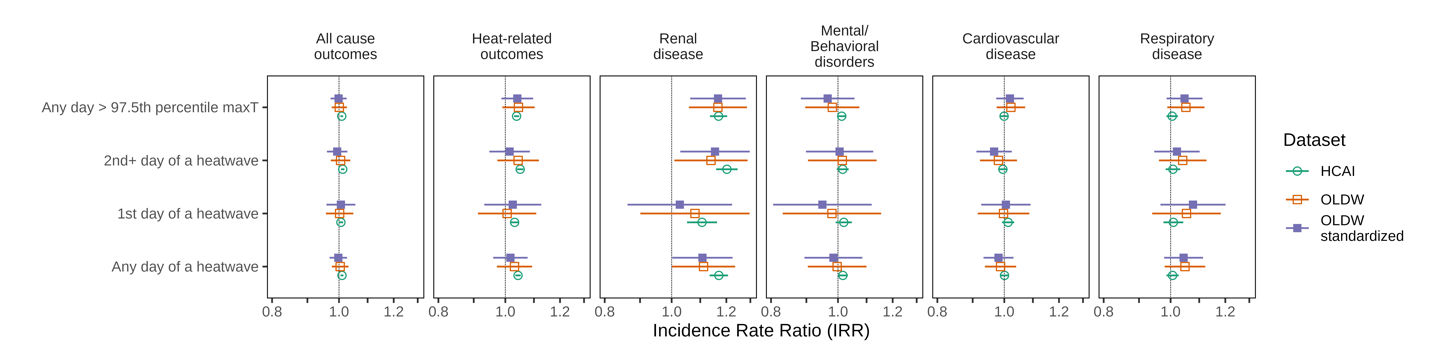


**eFigure 7**. IP incidence rate ratios for all ages by. Yaxis has a log scale. The ‘Any day of a heatwave’ category is what is plotted in Figure 1, panel B.

1. Altman DG, Bland JM. Interaction revisited: the difference between two estimates. *BMJ*. 2003;326(7382):219. doi:10.1136/bmj.326.7382.219 [↑](#footnote-ref-2)
2. Armstrong and Gasparrini 2013, <https://bmcmedresmethodol.biomedcentral.com/articles/10.1186/1471-2288-14-122> [↑](#footnote-ref-3)
